# Prenatal Vitamin D, Multivitamin, and Folic Acid Supplementation and Brain Structure in Children with ADHD and ASD Traits: The Generation R Study

**DOI:** 10.3390/nu17182979

**Published:** 2025-09-17

**Authors:** Daan van Rooij, Yuchan Mou, Tonya White, Trudy Voortman, Pauline W. Jansen, Jan K. Buitelaar

**Affiliations:** 1 Department of Experimental Psychology, Faculty Social Sciences, Utrecht University, 3584 CS Utrecht, The Netherlands; 2 Donders Center for Cognitive Neuroimaging, 6500 HB Nijmegen, The Netherlands; jan.buitelaar@radboudumc.nl; 3 Department of Medical Neuroscience, Radboud University Medical Centre, 6525 EN Nijmegen, The Netherlands; 4 Department of Epidemiology, Erasmus MC, University Medical Center Rotterdam, 3015 CN Rotterdam, The Netherlands; y.mou@erasmusmc.nl; 5 Department of Child- and Adolescent Psychiatry, Erasmus MC, University Medical Center Rotterdam, 3015 CN Rotterdam, The Netherlands; p.w.jansen@erasmusmc.nl; 6 The Generation R Study Group, Erasmus MC University Medical Center, 3015 CN Rotterdam, The Netherlands; 7 Section on Social and Cognitive Developmental Neuroscience, National Institute of Mental Health, Bethesda, MD 20814, USA; tonya.white@nih.gov; 8 Department of Psychology, Education and Child Studies, Erasmus Universiteit Rotterdam, 3062 PA Rotterdam, The Netherlands; trudy.voortman@erasmusmc.nl

**Keywords:** micronutrients, pregnancy, vitamin D, multivitamin supplements, ADHD, ASD, (s)MRI

## Abstract

Background/Objectives: Maternal vitamin supplementation (including folic acid, vitamin D, and multivitamin supplements) during pregnancy may lower the likelihood of neurodevelopmental disorders in offspring. This study examines the associations between maternal vitamin suppletion during pregnancy and morphological patterns in offsprings’ brain structure and traits of Autism Spectrum Disorder (ASD) and Attention-Deficit Hyperactivity Disorder (ADHD) in a large population-based study of child development. Methods: The study cohort included a total of 3937 children (aged 9–11) participating in the Generation R cohort in Rotterdam, the Netherlands. Maternal vitamin D and folateserum levels, multivitamin supplement use, and overall dietary quality (as assessed by the Food Frequency Questionnaire, FFQ) during pregnancy were used as predictors. T1 structural MRI scans were acquired and segmented using Freesurfer to assess brain morphometry. Cortical and subcortical brain volumes of children were separated into four independent components and used as mediators. ADHD and ASD traits, as measured by parent-completed questionnaires (Child Behavior CheckList and Social Responsiveness Scale, respectively) were used as outcome variables. Results: Results show that (1) maternal vitamin D, multivitamin supplementation, and better diet quality were associated with fewer ADHD or ASD traits in the offspring; (2) vitamin D and diet quality were associated with larger-volume childhood brain components; (3) larger-volume brain components were associated with fewer ADHD and ASD traits; (4) part of the association between dietary factors in pregnancy and offspring ADHD and ASD traits was mediated through the brain volumes of the children. Conclusions: Though all observed effect sizes were small, further population-based research should be performed to further delineate the effects of gestational multivitamin and vitamin D exposure and investigate whether this may be an avenue for preventive interventions.

## 1. Introduction

High-quality nutrition in mothers before conception and in fetuses prenatally has been shown to be critical for optimal fetal brain development. A growing body of evidence suggests that folic acid and vitamin D supplements, for instance, play a crucial role in brain development and function, and deficiencies in certain nutrients during pregnancy have been linked to worse physical and mental development in children, even long after birth [1]. Taken during pregnancy, multivitamin, vitamin D, and folic acid supplementation may help to prevent or lower the severity of offsprings’ internalizing mood disorders, such as anxiety and depression [2,3,4].

Relatively low availability of vitamins during critical periods of prenatal and early postnatal development have further been associated with an increased risk of neurodevelopmental disorders, including Autism Spectrum Disorder (ASD) and Attention-Deficit Hyperactivity Disorder (ADHD) [2,5,6].

A systematic review and meta-analysis of six prospective cohort studies in mostly European populations found an overall inverse association between maternal folic acid or multivitamin supplementation and children’s likelihood of ASD (relative risk, RR, for ASD of 0.64; 95% CI: 0.46, 0.90) [7]. Levine at al. [8], for instance, reported an association of maternal use of folic acid and multivitamin supplements in the periods before and during pregnancy with a 50% or larger reduction in likelihood of ASD in a cohort of more than 45,000 children at an age of 10 years. In [9], a 40% lower likelihood of ASD with intellectual disability was found in more than 270,000 mother–child pairs from population registers for mothers who used multivitamin supplements during pregnancy. Also, among very young children at high familial risk of ASD, those with a mother who used multivitamins before and/or in the first period of pregnancy were less likely to develop ASD, compared to those whose mothers did not use multivitamins [10]. Previous findings in the Generation R cohort also show that offspring of mothers with high vitamin D levels during pregnancy showed lower ASD traits at 6 years old [11]. Findings on the associations between dietary factors and/or vitamin use during pregnancy and likelihood of ADHD in the offspring are inconsistent [7], with some studies showing a significant link of folic acid and multivitamin use with a lower ADHD prevalence [12,13]. However, another population-based birth cohort found no such association [14].

Taken together, the associations of use of multivitamin/dietary supplements (including folic acid and vitamin D) with offspring neurodevelopmental disorders are not well understood [7]. Importantly, the overall quality and composition of a mother’s diet, as reflected in their general food patterns, may also contribute to ADHD and ASD traits in the offspring [15,16], but this has also hardly been accounted for in previous studies.

Moreover, the neurobiological pathways underlying the link between maternal dietary factors and offspring neurodevelopment remain largely uninvestigated. Previous studies indicate that maternal folic acid and vitamin D use and overall diet quality are associated with brain morphometry in offspring [17,18,19]. This was also the conclusion of a recent analysis using UK Biobank data, which indicated that the association between individuals’ dietary patterns and impulsive behavior was mediated through differences in brain structure [20].

Dietary factors may therefore affect the risk of neurodevelopmental disorders through differences in structural brain development, specifically by alterations in frontal–striatal pathways, which are strongly associated with neurodevelopmental disorders [21]. Currently, it is unclear which food components are more critical than others and what the role of the overall diet is, and it is also unclear to which extent early nutrition is associated with differences in brain structure and function in offspring and whether brain differences mediate offspring’s likelihood of neurodevelopmental characteristics. We expect that a similar pathway of structural brain morphometry may mediate the association of maternal pregnancy multivitamin, folic acid, and vitamin D use with offspring ADHD and ASD traits.

This study examines the associations of vitamin supplementation (specifically folic acid, vitamin D, and multivitamin supplements) with morphological patterns in offspring’s brain structure and traits of neurodevelopmental disorders (ASD and ADHD), while taking overall diet into account. Further, we test whether differences in the brain mediate the association between vitamin suppletion and ASD and ADHD traits in children.

Given this aim, we formulated four specific research questions and hypotheses which were investigated in the following order.

1. We investigated the association between maternal vitamin D, folate levels, and multivitamins and both ASD and ADHD traits in children. Our hypothesis was that supplementation with these vitamins during pregnancy would be associated with lower ASD and ADHD traits in children.
2. We investigated the association between maternal dietary supplements and structural brain morphometry in children. We expected that vitamin D, folate levels, and multivitamin use would all have significant associations with brain structure in the frontal–temporal and frontal–striatal structures, which are most associated with ASD and ADHD, respectively.
3. We investigated whether ASD and ADHD traits in children are associated with brain structure morphometry. We expected to replicate earlier findings of lower cortical thickness in frontal–temporal areas in ASD and frontal–striatal/parietal areas in ADHD.
4. Assuming that all the above stated effects were observed, we investigated whether the association between dietary supplements and ASD/ADHD traits is mediated through brain structure morphometry measures. We expected that part of the association between diet and neurodevelopmental disorders could be explained by differences in brain structure.

## 2. Methods

### 2.1. Sample Characteristics

We used data from the Generation R Study, a population-based prospective cohort that has followed approximately 10,000 children from fetal life into adulthood [22]. The study was established to identify early environmental and genetic determinants of normal and abnormal growth and development. The original study was approved by Ethics Committee of Erasmus UMC (protocol code MEC 198.782/2001/31; first date of approval: 9 December 2016). Detailed descriptions of the study design, data collection, and preprocessing procedures are available elsewhere [22,23,24]. For the present analysis, it is important to note that mothers were recruited during pregnancy (2002–2006), when questionnaires and blood sample data were collected at multiple time points. Their children have since been followed longitudinally, with behavioral and imaging data used in the current study obtained between 10 and 13 years of age.

The main predictors used for this study were maternal use of dietary vitamin D, folic acid, and multivitamin supplements during pregnancy. Information on use of maternal multivitamin supplements was based on a self-report questionnaire on the nutritional intake of mothers over the past three months, which was assessed in early pregnancy, directly after enrollment into the study (median, 13.5 weeks; range, 10.1–21.8) using the Food Frequency Questionnaire (FFQ) [25]. A pre-defined diet quality score was developed based on Dutch dietary guidelines, as previously described [26].

Folate serum levels and vitamin D serum levels were also obtained during early and mid-pregnancy, respectively (median gestational age of 13.07 and range of 12.07–14.64 for folate (first prenatal cohort visit) and median age of 20.36 and range of 19.93–20.93 for vitamin D, during second prenatal cohort visit). Details of the serum acquisition methods and details on the assessments can be found elsewhere for folate [27] and vitamin D [28], From here on, vitamin D will refer to the total 25OHD, the sum of 25-hydroxyvitamin D2 (25OHD2) and 25-hydroxyvitamin D3 (25OHD3) in plasma (nmol/L) [28]. Folate levels will refer to folate concentration in plasma (nmol/L) [27].

### 2.2. Behavioral Outcome Variables

The main outcomes for our models were ADHD and ASD traits in children. ADHD traits were measured by the parent-reported Child Behavior CheckList, using the DSM-oriented Attention-Deficit/Hyperactivity Problems scale T-score at the age 9 years (median age, 9.67; range, 9.54–9.81). The validated CBCL/6-18 is a 113-item parent-rated assessment of a child’s emotional and behavior problems [29], including the 11-item ADHD problem scale. ASD traits were measured by parent-reported the SRS-2 questionnaire, taken at the age 13 years (median age, 13.46; range, 13.32–13.59). The validated Social Responsiveness Scale (SRS) [30] is a quantitative measure of autistic traits in youth aged 4 to 18 years [30]. We used the overall total score of an abbreviated 18-item version of the SRS, which is highly correlated with the full version [4].

### 2.3. MRI Variables

Mediator variables included the cortical and subcortical brain volume of children at ages 9–11 (mean age, 10.12; sd = 0.59 years). These data were derived from 3 Tesla T_1_-weighted structural MRI scans following standard Freesurfer segmentation and averaged over both hemispheres, leading to a total of 43 included brain segmentations per subject. T_1_-weighted MRI data were centrally preprocessed and quality-controlled using a standard pipeline (see [4] for the sequence parameters, imaging protocol, and quality assessment of the images). ICA decomposition of these volumes was performed using the Infomax ICA algorithm, implemented using the icaimax package in R (R version 4.3.10). A total of 3737 Generation R participants had brain imaging data acquired at ages 9–11 and were included in the current analyses. Since not all participants with MRI data had complete information on all other variables (see availability in Table 1), we conducted Little’s MCAR test across all variables reported in Table 1. The results showed no significant effects, suggesting that missing MRI values were Missing Completely At Random (MCAR). Thus, these variables were unlikely to bias the statistical analyses. Therefore, all further analyses were performed using the maximum number of participants available for the specific variables included in each test.

### 2.4. Statistical Analyses

**Figure 1:**
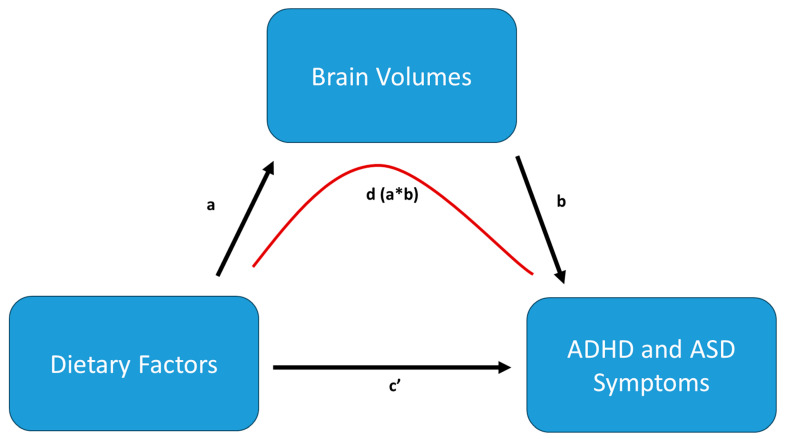
An example of the mediation model postulated above for illustrative purposes. The different paths correspond to each analysis step. Path a tests the association between dietary factors and brain volumes, path b the association between brain volumes and ADHD/ASD traits, path c’ the association between dietary factors and ADHD/ASD traits, and path d the indirect effect of dietary factors on ADHD/ASD mediated through brain volumes (a multiplied by b).

Given our hypotheses and aim to test the mediating role of brain structure in regulating the association between the dietary supplements and ASD/ADHD traits, our data analyses took the form of 4 sequential modeling steps, leading to a full multiple-mediation model (see Figure 1).

Model 1: Associations between dietary supplements during pregnancy and ADHD and ASD traits in children were analyzed using regression analyses with all supplements (vitamin D serum levels, folate serum levels, recent self-reported multivitamin use (yes/no), and overall diet quality) included as predictors and either total ADHD traits reported by the parental CBCL score or total ASD traits as reported by the parental SRS score as outcome measures (see Figure 1, path c’). For these and the following models, only subjects with available data on both the predictor and outcome variables were included. The resulting four models included child age (at MRI scan) and sex, and *p*-values were adjusted for multiple comparisons using false discovery rate (FDR) corrections. Importantly, all models including dietary variables were multivariate, incorporating all four dietary variables together. Thus, any reported associations reflect only the unique variance explained by each dietary variable.

Model 2: Brain data were segmented into 43 cortical and subcortical volumes. Given the large number of brain metrics, we first performed ICA decomposition of all brain regions into several underlying independent components as a fully data-driven data-reduction step. This allowed for the use of a far fewer number of the resulting independent components (ICs) as subsequent predictors and mediators in all statistical models.

Associations between dietary supplements during pregnancy and brain volumes were analyzed using a set of regression analyses with the dietary supplements (vitamin D serum levels, folate serum levels, self-reported multivitamin use (yes/no), and overall dietary quality) as predictors and each resulting brain IC as the outcome variable (see Figure 1, path a). The resulting four models accounted for child age and sex and were adjusted for multiple comparisons using FDR corrections.

Model 3: Associations between ADHD and ASD traits and brain volumes in children were analyzed using a set of regression models using the ADHD traits (reported by parental CBCL score) or total ASD traits (as reported by parental SRS) as outcome variables and each brain IC as predictors. These resulting models included child age and sex as additional variables and were corrected for multiple comparisons using FDR corrections (see Figure 1, path b).

Model 4: The final mediation models used the 3 supplements (vitamin D serum levels, folate serum levels, recent self-reported multivitamin use (yes/no), and overall dietary quality) as predictors, either total ADHD traits or total ASD traits as outcome measures, and the brain volume ICs as mediators. One multiple-mediation model was run for each predictor, using all brain ICs as mediators, with separate models for ADHD and ASD traits as dependent variables, respectively (see Figure 1, path d).

### 2.5. Exploratory Analyses of Additional Important Covariates

As stated above, all primary analyses were corrected for the effects of child age and sex, given potential differences between boys and girls in both brain development and the expression of ASD/ADHD traits.

Several other important covariates were identified that might have influenced the size and direction of our primary effects but were of no direct interest for our current study. Hence, a set of additional models is presented in the Supplementary Materials, showing the effects of birth weight and gestational age, maternal smoking and drinking during pregnancy, and maternal educational attainment and total household income.

Smoking and drinking were assessed during early, middle, and late pregnancy and coded based on the continuation of smoking (never/until pregnancy/during pregnancy) [31] and drinking (never drank/drank until pregnancy/drank occasionally during pregnancy/drank frequently during pregnancy) [32]. Maternal educational attainment (no education/lower/secondary education phase 1/secondary phase 2/higher phase 1/higher phase 2) and total household income (quantified in bins of EUR 200 increments) were assessed at enrollment [22]. Birth weight (grams) and gestational age at birth (weeks) were based on obstetric records of mothers [33].

Both smoking and drinking during pregnancy have been linked to adverse development of the fetus, and including these two factors ensured that any observed dietary effects were irrespective of overall lifestyle choices during pregnancy, which have been shown in other studies to potentially covary with dietary quality and supplement use [34]. Gestational age and birth weight of the child were used as a proxy for general developmental abnormalities during pregnancy and discounted that the overall physical health of the fetus may explain any association between diet and brain development later in life [35]. Lastly, maternal educational attainment and household income were used as a proxy of socio-economic status (SES). SES can be strongly associated with dietary choices, as well as brain measures, as also indicated in previous findings [36,37].

## 3. Results

### 3.1. Model 1: Dietary Supplements During Pregnancy and ADHD and ASD Traits in Children at Age 6 (Path C’)

The regression models in Table 2 indicate a marginally significant inverse association between maternal multivitamin use and ADHD (β = −0.48; t = 1.81; *p* < 0.07) and a significant association between maternal diet quality and ADHD (β = −0.30; t = −3.69; *p* < 0.001). The association reflects that use of multivitamins and higher diet quality is associated with lower levels of ADHD traits. Additionally, significant inverse associations between vitamin D levels and dietary quality and ASD are observed as well (β = −0.07, t = −2.64, and *p* < 0.008 and β = −0.056, t = −2.09, and *p* < 0.04, respectively), again reflecting that higher levels of vitamin D and higher dietary quality are associated with lower levels of ASD traits.

### 3.2. ICA Decomposition of Brain Segmentation

**Figure 2:**
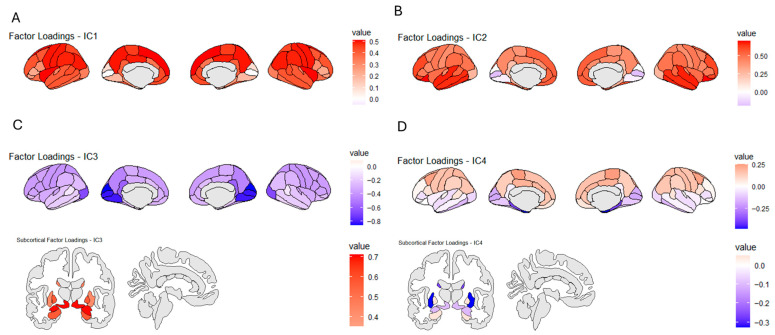
Results of the data-driven decomposition of segmented MRI data using Independent Component Analysis. Subsections (**A**–**D**) indicate the loadings of each brain segment in the four resulting independent components (ICs). (**A**). IC 1, defined by high factor loadings in the temporal/parietal areas. (**B**). IC 2, defined by high loadings in frontal and temporal areas. (**C**). IC 3, defined by negative loading in the occipital areas and high loadings in subcortical volumes. (**D**). IC 4, defined by negative loadings in the medial temporal, parahippocampal, and hippocampal regions.

ICA decompositions were first obtained for different numbers of independent components (ICs, N = 3–6). The final decomposition was subsequently selected based on biological interpretability and the non-redundancy of the resulting components, with a preference for the solution which included fewer factors. This resulted in the selection of a four-factor ICA decomposition, which is shown in Figure 2. Each IC consists of factor loadings in all of the 43 brain segments, with higher loading indicating a stronger contribution of these brain segments to the identity of each component. The first factor (IC1) in the decomposition is characterized by the highest relative factor loadings in the parietal cortex and temporal–parietal junction and will be referred to as the temporal/parietal component. Independent component 2 (IC2) is characterized by high loadings in the frontal and temporal cortex and will hence be referred to as the frontal–temporal IC. Independent component 3 (IC3) can be characterized by negative loading in the occipital cortex and high loadings in subcortical volumes and will be referred to as the subcortical IC. Independent component 4 (IC4) can be characterized by negative loadings in the medial temporal, parahippocampal, and hippocampal regions and will be referred to as the hippocampal IC. Full factor loadings for all ICs can be found in (Supplementary Materials S3).

### 3.3. Model 2: Dietary Supplements During Pregnancy and Brain Volumes in Children at Age 10 (Path a)

The regression models presented in Table 3 indicate a significant association between maternal vitamin D and brain volumes in the temporal/parietal IC (IC1) as well as the frontal–temporal IC (IC2) (β = 0.06, t = 2.23, and *p* = 0.03 and β = −0.08, t = 3.05, and *p* < 0.001, respectively). Additionally, significant associations between overall maternal diet quality and brain volume in the temporal/parietal IC (IC1), frontal–temporal (IC2), and subcortical IC (IC3) are observed (β = −0.06, t = 2.29, and *p* = 0.02, β = −0.08, t = −3.44, and *p* < 0.001, and β = 0.05, t = 2.15, and *p* = 0.03, respectively). Folate shows a positive association with volume in the subcortical IC (β = 0.06, t = 2.19, *p* = 0.03). Interestingly, all associations show a positive relation between dietary factors and brain volumes, except for the frontal–temporal IC2, for which higher vitamin D levels and better maternal dietary quality are associated with lower volumes.

### 3.4. Model 3: Associations Between Brain ICA Components and ADHD and ASD Traits in Children (Path b)

In Table 4, the regression models utilizing the brain ICs show that ADHD traits are negatively associated with brain volumes in the temporal/parietal and frontal–temporal ICs (β = −0.07, t = −3.78, and *p* < 0.001 and β = −0.05, t = −2.72, and *p* = <0.001, respectively) but positively associated with the subcortical IC (β = 0.10, t = 5.79, *p* < 0.001). ASD traits are negatively associated with the temporal/parietal and frontal–temporal ICs (β = −0.05, t = −2.79, and *p* = 0.01 and β = −0.08, t = −4.26, and *p* < 0.001, respectively) and positively associated with the subcortical IC (β = 0.06, t = 3.09, *p* < 0.001). These associations indicate that ADHD traits and ASD traits are associated with lower volumes in the temporal/parietal and frontal–temporal areas but higher volumes in the subcortical IC.

### 3.5. Model 4: Mediation of the Dietary Supplement Association with ADHD and ASD by Brain ICA Components (Path d)

The mediation models are presented in Table 5 and Table 6. The mediation models for ADHD traits (Table 5) indicate that the indirect effect of vitamin D on ADHD traits via the temporal/parietal IC1 is significant (mediation model 1; z = −2.67; *p* = 0.008), that the indirect effect of multivitamin use on ADHD traits via the temporal/parietal IC1 is significant (mediation model 2; z = −2.18; *p* = 0.029), and that the indirect effect of maternal dietary score on ADHD traits via the temporal/parietal IC1 is significant (mediation model 4; z = −2.51; *p* = 0.012).

The mediation models grouped for ASD traits (Table 6) indicate that the indirect effect of multivitamin use on ASD traits via the frontal–temporal IC2 is significant (t = −2.00; *p* = 0.045).

The full output for the multiple-mediation models including paths a–c, covariance matrices, and R^2^ values is shown in Supplementary Materials S2.

### 3.6. Exploratory Analyses of Key Covariates

In the Supplementary Materials, we report the effect of several important covariates on models 1–3, to preclude that any of our main findings are caused by spurious correlations with maternal substance use, socio-economic factors, or pregnancy health. Specifically, the below models were adjusted for child age, sex, gestational age, birth weight, prenatal smoking and drinking, maternal education, and household income.

The first supplementary model (see Supplementary Materials S1, SM Table S2) replicates model 1, assessing associations between prenatal diet and ADHD/ASD traits. Adjusted for the mentioned covariates, the primary dietary effects remain, though the association between dietary quality and ASD traits is no longer significant.

The second supplementary model (see Supplementary Materials S1, SM Table S3) replicates model 2, assessing associations between dietary factors and brain ICs, controlling for the same covariates. The primary associations between diet and brain volumes partially persist, though some dietary effects lose significance, likely due to reduced power. Specifically, the associations involving vitamin D and folate levels show similar beta values but no longer reach FDR-corrected significance.

The third supplementary model (see Supplementary Materials S1, SM Table S4) replicates model 3, examining associations between brain ICs and ADHD/ASD traits, controlling for the same covariates. The primary associations between brain volume ICs and ADHD/ASD traits largely remain unchanged.

To summarize across all these supplementary models, the previously observed links between prenatal diet, brain ICs, and ADHD/ASD traits remain largely consistent after adjusting for all these covariates. Given this stability and to maintain statistical power, multiple-mediation models only include correction of age and sex but not that of other covariates.

## 4. Discussion

Using a large population-based cohort, we show that multivitamin supplementation and better overall diet quality of the mother during pregnancy are associated with lower ADHD scores in children, whilst higher vitamin D serum levels and multivitamin supplementation are associated with lower ASD scores. Furthermore, we show that nutritional factors during pregnancy are associated with differences in brain volumes of offspring at ages 9–11. More specifically, vitamin D levels during pregnancy are associated with lower frontal–occipital and higher frontal–temporal volumes in children, whereas overall diet quality of mothers is associated with lower temporal/parietal and higher subcortical and frontal–temporal volumes in children. The mediation models indicate that the effects of maternal vitamin D levels, multivitamin supplementation, and dietary quality on ADHD traits in children are partly mediated through alterations in temporal and parietal brain volumes. Similarly, the effect of maternal multivitamin supplementation on ASD traits in children is partly mediated through changes in frontal–temporal brain volumes.

It must be noted that all effect sizes of the indirect effects observed in our mediation models represent only a small effect on eventual characteristics of either disorder. The R^2^ table (Supplementary Materials S2) indicates that dietary factors explain a small percentage of behavioral phenotype, with the indirect mediation through brain volumes again accounting for only a part of that effect (specifically, R^2^ values of the effect of dietary factors on behavior lie between 0.009 and 0.015, and the indirect effects of the brain ICs on behavior lie between 0.001 and 0.007). Hence, from these analyses, it is not directly observable what the clinical relevance of these effect sizes would be in practice.

Our finding that maternal vitamin D levels are associated with ASD traits in children corresponds with other cohort studies [38], as well as previous findings from the Generation R cohort [11]. Here we also show that vitamin D levels in mothers are linked to brain development in offspring. Similarly, our finding that multivitamin use in mothers is associated with lower ADHD levels in offspring is also in line with previous cohort findings [12], and our mediation models show that the association of multivitamin use with neurodevelopmental traits is mediated through frontal–occipital and frontal–parietal brain volumes.

Both prenatal vitamin D and multivitamin supplementation are linked to different aspects of brain development. The regulation of Brain-Derived Neurotropic Factor (BDNF) has been linked to vitamin levels [39,40,41], as has the regulation of neurotransmitter systems during brain development [42,43]. Both processes might explain the link between maternal diet and lower frontal–occipital and frontal–parietal volumes in ADHD and ASD, as decreased BDNF and dysregulation of neurotransmitter levels during pregnancy may both cause an overall disruption of cortical development. Additionally, vitamin levels have been associated with anti-inflammatory properties [44,45] and lower oxidative stress [46], which also aid in overall healthy brain development.

Although less direct evidence is available, it is plausible that overall dietary quality influences brain development via similar biological pathways, which is supported by some recent evidence showing the association between overall maternal dietary quality and brain development, cognition, and behavior in offspring [19,42,47,48,49,50].

Hence, our findings stress the likely importance of maternal nutrition in brain development. Although we did not directly test causal influences, these findings give a strong indication that multivitamin and vitamin D use might offer a small impact but statistically significantly lower the neurodevelopmental characteristics in offspring and that these effects are in part mediated by differences in brain volumes.

Contrary to our hypotheses, we found no significant unique effects of folate levels on ADHD or ASD traits, after correcting for the other dietary factors in our models. This may indicate that multivitamins and vitamin D are more important predictive factors for the development of ADHD and ASD characteristics than folate levels. It may be the case that the cohort overall had sufficiently high levels of folic acid supplementation and precluded us from observing a strong effect of this variable. It is of note that in contrast to in other countries, folic acid fortification of flour or other staples is not mandated in the Netherlands. Nevertheless, folic acid supplementation during pregnancy has been recommended in official government guidelines for over 30 years, which may have similarly led to a ceiling effect in the overall cohort. Nevertheless, previous analyses in the same cohort observed that higher maternal folate levels, as reported by maternal questionnaires, were associated with less internalizing and externalizing behavior in children [51]. Yet the average folate serum level observed in our sample (19.2 nmol/L) is much closer to that of the group from [51] which reported fewer childhood externalizing traits (23.5 nmol/L) than that of the group reporting more childhood externalizing problems (11.1 nmol/L). Besides the differences in folic acid assessment, the difference in these findings may also be explained by the fact that the [51] study did not incorporate multivitamin use, vitamin D levels, and overall dietary quality as covariates in its models, therefore potentially overestimating the effects of folic acid where these factors are covarying.

In addition to our main analyses, we report on several explorative models that corrected for potential covariates and other important factors in the Supplementary Materials (see Supplementary Materials S1). Adjusting for these covariates mostly did not alter the reported main associations between dietary factors and brain/behavior. The corrected models showed less power, which may have been due to the added variables in these models or because a part of the association between maternal diet and brain/cognition is confounded by socio-demographic factors or pregnancy health. However, we demonstrate that our main findings are robust and independent of the effects of maternal education, household income, maternal smoking and drinking during pregnancy, birth weight, and gestational age.

### Limitations

The first important caveat in our current analyses is that the Generation R birth cohort is a population-based cohort, where we have no influence over the dietary intake of participants. Hence, we cannot infer any causal links in any of our models that are based on observational data. Furthermore, regarding the distribution of dietary intake within our sample, it seems for some factors (for instance, folate serum levels), ceiling effects were present, making the currently studied variance non-informative for our outcome measures. The Generation R cohort may be subject to some selection bias (higher participation among higher-SES and Dutch-origin families) and attrition bias (loss to follow-up, particularly for the MRI data, especially in lower-SES and migrant groups), as described in previous publications [22,23,24]. Nevertheless, it remains a large, population-based, multi-ethnic sample, broadly reflecting the urban Dutch population. Thus, we have high confidence in the overall generalizability of our findings. Therefore, given these limitations, we can have even more confidence in the significant associations that we *do* observe, such as for multivitamin use, vitamin D, and overall dietary quality.

A second important caveat of the current research is the difficulty of dissociating covarying environmental and confounding factors during development, especially since many environmental and confounding factors tend to cluster and there can be difficulty in parsing the effect of individual environmental factors [52]. Our models will need to be expanded to investigate effects of other prenatal factors, as well as the wider environment during pregnancy and early life development. This includes factors like environmental stressors in utero and postnatally, exposure to toxins, and socio-demographic factors that were not captured by our measures of socio-economic status. Finally, part of these effects may be mediated by or driven by genetic factors in the mothers and the offspring. The dissociation of all these potential confounding factors goes beyond the scope of the current paper, but we wish to emphasize that many factors may also influence brain development and mental health beyond the ones studied in the current analyses, and our future efforts will be directed towards unraveling the independent effects of as many of these factors as possible.

## 5. Conclusions

Taken as a whole, our research shows several important findings. Firstly, we show associations between maternal dietary factors during pregnancy and variance in brains and behavior of offspring many years later. Second, we also show that we can dissociate which dietary factors and which brain ICs are associated with traits of different neurodevelopmental disorders. So both dietary factors and brain volume patterns seem to be specific for either ADHD or ASD. Lastly, we show that through these mediation models, it is likely that some of the influence of diet is mediated through structural brain development, indicating a potential causal pathway through which the influence of vitamin and other dietary factors may influence behavioral outcomes.

This highlights the importance of dietary programs and other preventative interventions not only in promoting physical health but also in potentially ameliorating effects of different (neuro)psychiatric disorders.

Our current results suggest that further research into the direct causal nature of specifically multivitamin and vitamin D supplementation and improved general maternal health should be performed on a populational level. We expect that a focus on education about diet and healthy living may provide vast benefits for mental health in the long term.

## Figures and Tables

**Table 1:** Participant characteristics and data availability.

	N Available	Mean or %	SD	Min.	Max.
Child age during MRI scan (years)	3737 (1951 males)	10.12	0.59	8.50	12.00
ASD traits (SRS overall score)	3070	4.93	3.83	0	46.00
ADHD traits (CBCL ADHD subscale T-score)	3334	52.99	5.03	50	80
Folate serum concentration during pregnancy (nmol/L)	2571	19.21	9.10	3.70	45.30
Vitamin D serum concentration during pregnancy (nmol/L)	3072	56.35	31.53	1.50	161.00
Multivitamin use during pregnancy (self-reported)	2930	34% yes	-	-	
Diet quality score during pregnancy (FFQ)	2709	7.75	1.57	1.55	12.99

**Table 2:** Regression outcomes of the association between dietary supplementation during pregnancy on ADHD and ASD traits in children, corrected for age and sex. β values indicate standardized beta coefficients, except for categorical variables, indicated with *, where unstandardized b values are depicted. Bold values indicate significant effects (FDR-corrected).

	ADHD Traits			ASD Traits		
	Beta (β)	t Value	*p* Value	Beta (β)	t Value	*p* Value
**Vitamin D (serum)**	−0.03	−1.09	0.27	**−0.07**	**−2.64**	**<0.008**
**Folate (serum)**	−0.01	−0.91	0.36	−0.03	−0.50	0.35
**Multivitamin use**	−0.47 *	**1.81**	**0.07**	−0.39 *	1.76	0.08
**Diet score pregnancy**	**−0.09**	**−3.68**	**<0.001**	**−0.05**	**−2.09**	**<0.04**
**Child age (year)**	0.01	0.55	0.577	0.00	0.32	0.975
**Sex (1 = male; 2 = female)**	−0.34 *	−1.44	0.150	−0.70 *	**−3.35**	**<0.001**

**Table 3:** Regression outcomes of the association between dietary supplementation during pregnancy on independent brain components in children, according to models including age and sex. β values indicate standardized beta coefficients, except for categorical variables, indicated with *, where unstandardized b values are depicted. Bold values indicate significant effects (*p*-values were FDR-corrected).

	IC1:	Temporal/Parietal			IC2:	Frontal–Temporal		
	Beta (β)	Std error	t value	*p* value	Beta (β)	Std error	t value	*p* value
**Vitamin D**	**0.06**	**0.00**	**2.23**	**0.03**	**−0.08**	**0.00**	**−3.05**	**<0.001**
**Folate**	0.01	0.00	0.42	0.68	0.01	0.00	0.49	0.62
**Multivitamin use**	0.04 *	0.05	1.46	0.14	−0.04 *	0.05	−1.52	0.13
**Diet score pregnancy**	**0.06**	**0.02**	**2.29**	**0.02**	**−0.08**	**0.02**	**−3.44**	**<0.001**
**Child age (year)**	0.03	0.04	1.16	0.25	**0.09**	**0.04**	**3.89**	**<0.001**
**Sex (1 = male; 2 = female)**	−0.14 *	**0.05**	**−5.91**	**<0.001**	0.23 *	**0.05**	**9.82**	**<0.001**
	**IC3:**	**Subcortical**			**IC4:**	**Hippocampal**		
	Beta (β)	Std error	t value	*p* value	Beta (β)	Std error	t value	*p* value
**Vitamin D**	0.02	0.00	0.71	0.48	−0.04	0.00	−1.56	0.12
**Folate**	**0.06**	**0.00**	**2.19**	**0.03**	0.04	0.00	1.67	0.10
**Multivitamin use**	−0.01 *	0.05	−0.34	0.73	0.00 *	0.05	−0.20	0.84
**Diet score pregnancy**	**0.05**	**0.01**	**2.15**	**0.03**	0.04	0.02	1.46	0.14
**Child age (year)**	**0.05**	**0.04**	**2.02**	**0.04**	**−0.08**	**0.04**	**−3.50**	**<0.001**
**Sex (1 = male; 2 = female)**	−0.36 *	**0.04**	**−15.83**	**<0.001**	0.12 *	**0.05**	**5.13**	**<0.001**

**Table 4:** Regression outcomes of associations between brain independent components and ADHD and ASD traits in children. β values indicate standardized beta coefficients, except for categorical variables, indicated with *, where unstandardized b values are depicted. Bold values indicate significant effects (*p*-values were FDR-corrected).

	ADHD			ASD		
	Beta (β)	t Value	*p* Value	Beta (β)	t Value	*p* Value
**IC1 (temporal/parietal)**	**−0.07**	**−3.78**	**<0.001**	**−0.05**	**−2.79**	**0.01**
**IC2 (frontal–temporal)**	**−0.05**	**−2.72**	**<0.001**	**−0.08**	**−4.26**	**<0.001**
**IC3 (subcortical)**	**0.10**	**5.79**	**<0.001**	**0.06**	**3.09**	**<0.001**
**IC4 (hippocampal)**	−0.01	−0.58	0.56	−0.01	−0.63	0.53
**Child age (year)**	**−0.11**	**−5.49**	**<0.001**	−0.12	−6.09	**<0.001**
**Sex (1 = male; 2 = female)**	0.00 *	−0.11	0.92	0.02 *	1.07	0.28

**Table 5:** Mediation models indicating the indirect effects (mediated through brain ICs) of dietary factors on ADHD, corrected for age and sex. Bolded values indicate significant effects.

**Mediation Model 1**					
**Vitamin D > ADHD**	Effect (path)	Estimate (b)	std. error	z value	*p* value (fdr)
IC 3: subcortical	Indirect 1 (d1)	0	0	−1.26	0.20
IC2: frontal–temporal	Indirect 2 (d2)	0	0	−1.31	0.19
IC1: temporal/parietal	**Indirect 3 (d3)**	**−0.001**	**0**	**−2.67**	**0.01**
IC4: hippocampal	Indirect 4 (d4)	0	0	−0.43	0.66
	Total (c)	−0.006	0.003	−1.66	0.09
**Mediation model 2**					
Folic acid > ADHD	Effect (path)	Estimate (b)	std. error	z value	*p* value (fdr)
IC 3: subcortical	Indirect 1 (d1)	0.00	0.00	−1.11	0.27
IC2: frontal–temporal	Indirect 2 (d2)	0.00	0.00	−1.66	0.10
IC1: temporal/parietal	Indirect 3 (d3)	0.00	0.00	−1.81	0.07
IC4: hippocampal	Indirect 4 (d4)	0.00	0.00	−0.68	0.50
	Total (c)	**−0.03**	**0.01**	**−2.27**	**0.02**
**Mediation model 3**					
Multivitamin use > ADHD	Effect (path)	Estimate (b)	std. error	z value	*p* value (fdr)
IC 3: subcortical	Indirect 1 (d1)	−0.01	0.011	−0.96	0.34
IC2: frontal–temporal	Indirect 2 (d2)	−0.02	0.015	−1.38	0.17
IC1: temporal/parietal	**Indirect 3 (d3)**	**−0.04**	**0.02**	**−2.18**	**0.03**
IC4: hippocampal	Indirect 4 (d4)	−0.004	0.008	−0.44	0.66
	Total (c)	0.05	0.21	0.25	0.79
**Mediation model 4**					
Diet score pregnancy > ADHD	Effect (path)	Estimate (b)	std. error	z value	*p* value (fdr)
IC 3: subcortical	Indirect 1 (d1)	−0.006	0.00	−1.52	0.127
IC2: frontal–temporal	Indirect 2 (d2)	−0.008	0.00	−1.50	0.133
IC1: temporal/parietal	**Indirect 3 (d3)**	**−0.015**	**0.00**	**−2.51**	**0.012**
IC4: hippocampal	Indirect 4 (d4)	−0.001	0.00	−0.45	0.648
	**Total (c)**	**−0.25**	**0.06**	**−4.10**	**0**

**Table 6:** Mediation models indicating the indirect effects (mediated through brain ICs) of dietary factors on ASD, corrected for age and sex. Bolded values indicate significant effects.

**Mediation model 5.**					
**Vitamin D > ASD**	Effect (path)	Estimate (b)	std. error	z value	*p* value (fdr)
IC 3: subcortical	Indirect 1 (d1)	0	0	−0.21	0.83
IC2: frontal–temporal	Indirect 2 (d2)	0	0	−1.86	0.06
IC1: temporal/parietal	Indirect 3 (d3)	0	0	−0.11	0.91
IC4: hippocampal	Indirect 4 (d4)	0	0	−0.28	0.77
	**Total (c)**	**−0.01**	**0.00**	**−4.76**	**0**
**Mediation model 6**					
Folic acid > ASD	Effect (path)	Estimate (b)	std. error	z value	*p* value (fdr)
IC 3: subcortical	Indirect 1 (d1)	0.00	0.00	−0.07	0.95
IC2: frontal–temporal	Indirect 2 (d2)	0.00	0.00	−1.60	0.11
IC1: temporal/parietal	Indirect 3 (d3)	0.00	0.00	−0.45	0.65
IC4: hippocampal	Indirect 4 (d4)	0.00	0.00	−1.33	0.18
	**Total (c)**	**−0.03**	**0.01**	**−3.11**	**<0.001**
**Mediation model 7**					
Multivitamin use > ASD	Effect (path)	Estimate (b)	std. error	z value	*p* value (fdr)
IC 3: subcortical	Indirect 1 (d1)	−0.001	0.00	−0.27	0.78
IC2: frontal–temporal	**Indirect 2 (d2**)	**−0.03**	**0.01**	**−2.00**	**0.04**
IC1: temporal/parietal	Indirect 3 (d3)	−0.018	0.01	−1.26	0.21
IC4: hippocampal	Indirect 4 (d4)	0	0.00	−0.06	0.95
	**Total (c)**	0.093	0.19	0.46	0.64
**Mediation model 8**					
Diet score pregnancy > ASD	Effect (path)	Estimate (b)	std. error	z value	*p* value (fdr)
IC3: subcortical	Indirect 1 (d1)	0	0.00	0.02	0.98
IC2: frontal–temporal	Indirect 2 (d2)	−0.008	0.00	−1.56	0.11
IC1: temporal/parietal	Indirect 3 (d3)	−0.004	0.00	−1.06	0.29
IC4: hippocampal	Indirect 4 (d4)	−0.001	0.00	−0.56	0.57
	**Total (c)**	**−0.19**	**0.05**	**−3.87**	**0**

## Data Availability

Data available on request due to legal restrictions: requests can be addressed to the Generation R Management Team (generationr@erasmusmc.nl). Data sharing within the Generation R cohort is governed by Data Use Agreements (DUA) and Data Transfer Agreements (DTA), ensuring adherence to relevant laws and regulations.

## Supplementary Materials

The following supporting information can be downloaded at https://www.mdpi.com/article/10.3390/nu17182979/s1. Supplementary Materials S1: Covariate analyses; Supplementary Materials S2: Mediation models; Supplementary Materials S3: ICA factor loading.
