# Supplementary material for: Prenatal Vitamin D, Multivitamin, and Folic Acid Supplementation and Brain Structure in Children with ADHD and ASD Traits: The Generation R Study
Source: Nutrients. 2025 Sep 17;17(18):2979. doi: 10.3390/nu17182979 (PMC12472298; doi:10.3390/nu17182979)
Supplement: Supplementary file 1 [file nutrients-17-02979-s001.zip › Manuscript_gen-r_diet_supplementary_part1_covariate analyses_proof_DvR.pdf]

Prenatal vitamin D, multivitamin and folic acid supplementation and brain structure in children with ADHD and ASD traits

Supplementary materials 1.

In these supplementary materials, we present: the full descriptive statistics for all variables, including the covariates not presented in the main manuscript. Secondly, we present the outcomes of models 1 – 3 from the main manuscript, corrected for this set of additional variables.

SI Table 1: Descriptive statistics:

	**ASD t–score (SRS)**	**ADHD t–score (CPRS)**	**Folic Acid (serum)**	**Vitamin D (serum)**	**Dietary quality pregnancy (FFQ self report)**	**Multivitamin supplementation pregnancy (self report)**	**Educational attainment mother**	**Household income**	**Maternal drinking (FFQ self report)**	**Maternal smoking**	**Birth weight child (gr)**	**Gestational Age child (wks)**
N	3070	3334	2599	3113	2745	2972	3655	3049	3245	3426	9662	9666
Mean	4.93	52.99	19.21	56.35	7.76	0.34	4.55	10.27	2.13	1.36	3384	39.69
Standard deviation	3.83	5.03	9.1	31.53	1.58	0.47	1.22	2.81	1.06	0.71	589	2.124

SI Table 2: Model 1. Associations between dietary supplements during pregnancy and ADHD and ASD traits in children (corrected for birth weight and gestational age, maternal smoking and drinking during pregnancy, and maternal educational attainment and household income).

**ADHD**			
	Beta (B)	t–value	p–value
Vitamin D	−0.02	−0.51	0.61
Folic acid	−0.01	−0.41	0.69
Diet score pregnancy	**−0.06**	**−2.12**	**0.03**
Multivitamin use	**0.53**	**1.90**	**0.06**
Sex (1=male, 2=female)	−0.45	−1.75	0.08
Gestational Age child (wks)	−0.05	−1.36	0.17
Birth weight child (gr)	−0.04	−1.24	0.22
Maternal smoking	**0.08**	**2.80**	**0.01**
Maternal drinking (FFQ self report)	−0.03	−0.85	0.40
Household income	0.00	−0.04	0.97
Educational attainment mother	**−0.10**	**−2.94**	**0.00**
**ASD**			
	Beta (B)	t–value	p–value
Vitamin D	**−0.06**	**−2.03**	**0.04**
Folic acid	0.01	0.36	0.72
Diet score pregnancy	−0.03	−0.90	0.37
Multivitamin use	0.27	1.23	0.22
Sex (1=male, 2=female)	**−0.59**	**−2.85**	**0.00**
Gestational Age child (wks)	−0.02	−0.59	0.55
Birth weight child (gr)	−0.06	−1.67	0.10
Maternal smoking	0.00	−0.03	0.98
Maternal drinking (FFQ self report)	−0.01	−0.24	0.81
Household income	−0.02	−0.61	0.55
Educational attainment mother	**−0.07**	**−2.03**	**0.04**

SI Table 3: Model 2. Associations between dietary supplements during pregnancy and brain volumes (corrected for birth weight and gestational age, maternal smoking and drinking during pregnancy, and maternal educational attainment and household income).

	IC1					IC2				
	Estimate	Standardized beta	Std. Error	t–value	p–value	Estimate	Standardized beta	Std. Error	t–value	p–value
Vitamin D	0.00	––0.03	0.00	––1.04	0.30	0.00	––0.01	0.00	–0.50	0.61
Folic acid	0.00	0.03	0.00	1.15	0.25	0.00	0.02	0.00	0.67	0.50
Diet score pregnancy	**–0.03**	**–0.05**	**0.02**	**–1.92**	**0.05**	0.02	0.03	0.02	1.05	0.29
Multivitamin use	–0.06	–0.03	0.05	–1.09	0.28	–0.01	–0.01	0.05	–0.24	0.81
Sex (1=male, 2=female)	**0.41**	**0.21**	**0.05**	**8.07**	**0.00**	**–0.62**	**–0.32**	**0.05**	**–13.39**	**0.00**
Child Age (yr)	**0.17**	**0.10**	**0.04**	**4.02**	**0.00**	0.06	0.04	0.04	1.60	0.11
Gestational Age child (wks)	**0.04**	**0.07**	**0.02**	**2.18**	**0.03**	–0.03	–0.05	0.02	–1.77	0.08
Birth weight child (gr)	**0.00**	**–0.09**	**0.00**	**–2.81**	**0.00**	**0.00**	**0.26**	**0.00**	**8.67**	**0.00**
Maternal smoking	–0.04	–0.03	0.04	–0.98	0.33	–0.01	0.00	0.04	–0.19	0.85
Maternal drinking (FFQ self report)	–0.02	–0.02	0.03	–0.72	0.47	–0.03	–0.03	0.02	1.22	0.22
Household income	**–0.03**	**–0.08**	**0.01**	**–2.52**	**0.01**	**0.02**	**0.06**	**0.01**	**1.99**	**0.05**
Educational attainment mother	–0.05	–0.05	0.03	–1.52	0.13	0.02	0.02	0.03	0.50	0.61
	IC3					IC4				
	Estimate	Standardized beta	Std. Error	t–value	p–value	Estimate	Standardized beta	Std. Error	t–value	p–value
Vitamin D	0.00	0.04	0.00	1.40	0.16	**0.00**	**–0.06**	**0.00**	**–2.10**	**0.04**
Folic acid	0.00	0.01	0.00	0.46	0.64	0.00	0.04	0.00	1.43	0.15
Diet score pregnancy	**0.03**	**0.05**	**0.02**	**1.81**	**0.07**	0.00	–0.01	0.02	–0.19	0.85
Multivitamin use	0.06	0.03	0.05	1.18	0.24	–0.02	–0.01	0.06	–0.39	0.70
Sex (1=male, 2=female)	**–0.26**	**–0.14**	**0.05**	**–5.17**	**0.00**	**0.27**	**0.13**	**0.05**	**5.14**	**0.00**
Child Age (yr)	0.06	0.03	0.04	1.30	0.20	**–0.15**	**–0.09**	**0.05**	**–3.28**	**0.00**
Gestational Age child (wks)	–0.02	–0.04	0.02	–1.21	0.23	0.01	0.02	0.02	0.71	0.48

Birth weight child (gr)	**0.00**	**0.06**	**0.00**	**1.95**	**0.05**	0.00	0.06	0.00	1.81	0.07
Maternal smoking	**−0.09**	**−0.06**	**0.04**	**−2.25**	**0.02**	−0.08	−0.05	0.04	−1.89	0.06
Maternal drinking (FFQ self report)	**−0.06**	**−0.07**	**0.03**	**2.31**	**0.02**	−0.03	−0.03	0.03	1.02	0.31
Household income	0.00	0.00	0.01	−0.04	0.97	0.01	0.02	0.01	0.53	0.60
Educational attainment mother	−0.01	−0.01	0.03	−0.17	0.87	0.03	0.03	0.03	1.01	0.31

SI Table 4: Model 3. Association between ADHD and ASD traits and brain volumes in children (corrected for birth weight and gestational age, maternal smoking and drinking during pregnancy, and maternal educational attainment and household income).

	**ADHD**				**ASD**			
	Estimate	Std. Error	t–value	p–value	Estimate	Std. Error	t–value	p–value
IC1 (frontal–occipital)	**−0.25**	**0.12**	**−2.18**	**0.03**	−0.08	0.09	−0.89	0.38
IC2 (frontal parietal)	−0.12	0.11	−1.13	0.26	**−0.25**	**0.09**	**−2.87**	**<.001**
IC3 (subcortical)	**0.43**	**0.11**	**4.02**	**<.001**	**0.14**	**0.09**	**1.59**	**0.11**
IC4 (hippocampal)	0.00	0.10	0.00	1.00	0.01	0.08	0.11	0.91
Sex	**−0.89**	**0.23**	**−3.86**	**<.001**	**−0.95**	**0.18**	**−5.20**	**<.001**
Gestational Age child (wks)	−0.09	0.07	−1.34	0.18	−0.08	0.05	−1.56	0.12
Birth weight child (gr)	0.00	0.00	0.55	0.58	0.00	0.00	−0.33	0.74
Maternal smoking	**0.66**	**0.16**	**4.21**	**<.001**	−0.05	0.13	−0.43	0.66
Maternal drinking (FFQ self report)	−0.06	0.11	−0.53	0.60	−0.04	0.08	−0.44	0.66
Household income	**−0.17**	**0.05**	**−3.56**	**<.001**	**−0.12**	**0.04**	**−3.03**	**<.001**
Educational attainment mother	0.03	0.13	0.23	0.82	**−0.24**	**0.10**	**−2.39**	**0.02**
